# Effect of MDI Actuation Timing on Inhalation Dosimetry in a Human Respiratory Tract Model

**DOI:** 10.3390/ph15010061

**Published:** 2022-01-04

**Authors:** Mohamed Talaat, Xiuhua Si, Jinxiang Xi

**Affiliations:** 1 Department of Biomedical Engineering, University of Massachusetts, Lowell, MA 01854, USA; Mohamed_Talaat@student.uml.edu; 2 Department of Aerospace, Industrial, and Mechanical Engineering, California Baptist University, Riverside, CA 92504, USA; asi@calbaptsit.edu

**Keywords:** metered dose inhaler (MDI), press-and-breathe, actuation–inhalation coordination, dispersion, orifice, high-speed imaging, polydisperse distribution

## Abstract

Accurate knowledge of the delivery of locally acting drug products, such as metered-dose inhaler (MDI) formulations, to large and small airways is essential to develop reliable in vitro/in vivo correlations (IVIVCs). However, challenges exist in modeling MDI delivery, due to the highly transient multiscale spray formation, the large variability in actuation–inhalation coordination, and the complex lung networks. The objective of this study was to develop/validate a computational MDI-releasing-delivery model and to evaluate the device actuation effects on the dose distribution with the newly developed model. An integrated MDI–mouth–lung (G9) geometry was developed. An albuterol MDI with the chlorofluorocarbon propellant was simulated with polydisperse aerosol size distribution measured by laser light scatter and aerosol discharge velocity derived from measurements taken while using a phase Doppler anemometry. The highly transient, multiscale airflow and droplet dynamics were simulated by using large eddy simulation (LES) and Lagrangian tracking with sufficiently fine computation mesh. A high-speed camera imaging of the MDI plume formation was conducted and compared with LES predictions. The aerosol discharge velocity at the MDI orifice was reversely determined to be 40 m/s based on the phase Doppler anemometry (PDA) measurements at two different locations from the mouthpiece. The LES-predicted instantaneous vortex structures and corresponding spray clouds resembled each other. There are three phases of the MDI plume evolution (discharging, dispersion, and dispensing), each with distinct features regardless of the actuation time. Good agreement was achieved between the predicted and measured doses in both the device, mouth–throat, and lung. Concerning the device–patient coordination, delayed MDI actuation increased drug deposition in the mouth and reduced drug delivery to the lung. Firing MDI before inhalation was found to increase drug loss in the device; however, it also reduced mouth–throat loss and increased lung doses in both the central and peripheral regions.

## 1. Introduction

Assessment of the therapeutic efficacy of oral inhalation drug products (OIDPs), such as metered-dose inhaler (MDI) formulations, requires accurate knowledge of their deposition in the targeted areas [1,2]. Although SPECT/CT can visualize the in vivo drug depositions, its application in studying inhalation drug delivery is limited due to high cost, radiation risks, and ethical issues [3,4,5,6]. Moreover, clinical trials of OIDPs with either lab animals or human subjects can take years to complete [7,8,9]. Surrogate computational fluid dynamics (CFD) simulations have become increasingly important in providing comparable results and deposition details [10,11]. However, an accurate CFD predicted dosimetry requires a precise representation of the drug properties, respiratory geometry, and breathing condition. Moreover, significant variability exists among individuals in respiratory structures, inhalation patterns, disease severity, and thus the MDI dose distributions, which are critical in developing reliable in vitro/in vivo correlation (IVIVC) [12,13,14]. The implications of such variability are particularly important in the selection and assessment of regulatory options. Given the rising demand for the knowledge of drug deposition and its variability, there is a clear need for improved CFD models that can precisely consider the details in the device, patient, and their interactions [15,16].

Many factors make the numerical investigation of MDI delivery to the human lung challenging [17,18,19,20,21]. These include the limited knowledge of MDI actuation and droplet generation, the difficulties in developing physiologically accurate lung geometries, and the large variability in patient–device interactions, including the frequent error reported in MDI usages: the discoordination between device triggering and inhalation [22,23,24]. The MDI actuation occurs within a very short period (0.1–0.2 s), and the spray droplets exit the nozzle at high speeds [25,26]. Due to the highly transient nature, the behaviors of the spray cloud and individual droplets discharged from the MDI are still not fully understood [27,28]. Schroeter et al. investigated the effects of MDI formulation variables on lung dosimetry and observed a direct correlation between predicted lung dose and fine particle fraction [29]. Liu et al. measured the droplet size distribution by using a laser light-scattering system (Sympatec) in MDI products with both chlorofluorocarbon (CFC) and hydrofluoroalkane (HFA) propellants [30]. They also measured the droplet speeds 3 and 6 cm downstream of the mouthpiece, using a phase Doppler anemometry (PDA). The exiting speeds at the nozzle orifice were not measured, which is needed when the effects of MDI–patient interactions are to be evaluated.

Visualization of MDI actuation and aerosol formation has been attempted by using high-speed camera, laser diffraction, Schlieren optical imaging, and phase-contrast X-ray imaging [31,32,33,34,35,36,37]. With a laser and high-speed camera, Smyth et al. observed asymmetrical and ellipsoid spray cross-sections in the vertical direction [38]. Mason-Smith et al. recorded the spray plume development in the near-nozzle region at 5 µm spatial and 0.184 ms temporal resolution, using X-ray radiography; they observed that the spray flow reached steady conditions around 30 ms after the actuation of HFA MDIs [34]. It is noted, however, that the MDI spray was discharged into a free space in most previous visualization studies, which could be different from the spray plume when released into a confined space. To characterize this difference, McKiernan released the MDI spray into a ventilated mouth–throat cast and visualized the plume formation by using both the phase-contrast X-ray and Schlieren imaging [32]. They reported an apparent counter-flow pattern of the aerosol cloud in the front-upper oral cavity [32].

To understand the transport and deposition of the MDI droplets in the respiratory tract, an integrated MDI–mouth–lung model is needed. Computational fluid dynamics (CFD) studies have demonstrated that the behaviors were different between spray aerosols discharged into an open space and a confined oral cavity [39,40]. The MDI spray plume angle can be significantly smaller in the presence of an inhalation flow compared to no flow conditions [41]. As a result, it has been suggested that plume geometry tests should be conducted under breathing conditions during drug delivery, even though doing so is still impractical when using current techniques [41]. To appropriately evaluate the MDI delivery, it also requires a lung model with sufficient geometrical details. Kleinstreuer et al. simulated droplet behavior and fates in an MDI and spacer connected to the upper airway geometry and reported improved bronchiolar dosing with the use of FHA, smaller valve orifice, and spacer [42]. The airway geometry in the above study extended to the trachea only, and thus could not differentiate the dosimetry in the central and peripheral airways. To estimate drug dosimetry in different regions of the lung, Longest et al. compared aerosol deposition from MDI and DPI (dry powder inhaler) in a stochastic individual path model, with each lobe having a single path extending to B15 [39]. This model has the advantage of relatively small mesh size and reasonable accuracy in capturing regional deposition (lobar, central, and peripheral) [43]. To this aim, the heterogeneity of lung branching and ventilations, along with the associated aerosol transport and deposition, had to be sacrificed.

As we stated above, lack of coordination between device triggering and breathing is one of the most frequent mistakes when using press-and-breathe MDIs [44,45]. Patients who have neuromuscular, cognitive, or respiratory impairments will have difficulties in achieving a required actuation–inhalation synchronization and thus may not derive full benefits from press-and-breathe MDIs [46]. Farkas et al. numerically investigated delayed MDI actuation on lung deposition in a model that combined a 3D mouth–throat model with a 1D stochastic whole lung and reported that late actuation led to lower lung doses [24]. Because droplet deposition in a given lung generation was computed by using analytical equations rather than a physical geometry, local (or cellular level) dosimetry could not be computed.

The objective of this study is to develop an integrated MDI–mouth–lung model extending to G9 and use it to investigate the effects of MDI actuation time on inhalation dosimetry. Specific aims are to (1) reversely identify the spray discharging velocity based on PDA-measured velocities 3 and 6 cm downstream of the mouthpiece, (2) record the temporal and spatial evolution of MDI droplet cloud, (3) examine the airflow and droplet dynamics in the control case (triggering at 0.63 s) in three phases (i.e., discharging, dispersion, and inhalation), (4) quantify the drug dosimetry on a regional and local basis for the control case, and (5) evaluate the impact of earlier and delay MDI triggering on inhalation dosimetry.

## 2. Results

### 2.1. High-Speed Imaging of MDI Releasing

**Figure 1:**
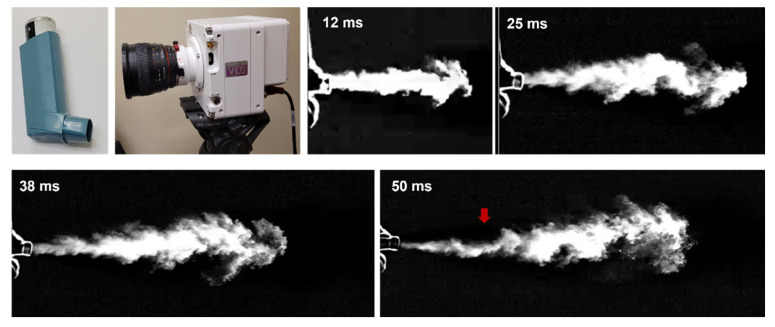
High-speed imaging of MDI releasing.

Figure 1 shows the spatial and temporal evolutions of the MDI aerosol plume recorded by a high-speed camera (Phantom VEO) at an acquisition rate of 4000 fps. A typical jet flow was captured that emanated from the nozzle and mouthpiece (12 ms). It became increasingly dispersed by mixing with the surrounding air and gradually slowed down with time (25–50 ms). Due to gravity, the jet slightly bent down (red arrow) at 50 ms, indicating that there existed slow-moving droplets, whose gravitational settling became non-negligible relative to their advection.

### 2.2. Measurement-Based Computational Platform for MDI Delivery

#### 2.2.1. Integrated MDI–Mouth–Lung Model and Waveforms

**Figure 2:**
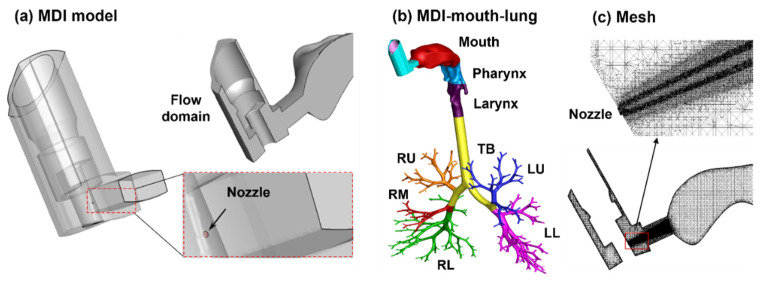
Computational model: (**a**) metered-dose inhaler (MDI) with an aerosol-exiting nozzle, (**b**) the integrated MDI–mouth–lung model, and (**c**) multi-block computational mesh with refined cells downstream of the nozzle. The airway model was divided into mouth, pharynx, larynx, tracheobronchial region (TB), and five lung lobes.

The MDI model geometry was developed in SolidWorks, based on an albuterol MDI (Figure 1). The nozzle had a diameter of 0.5 mm (Figure 2a, right lower panel). The MDI geometry was connected to the mouth opening of a mouth–lung model previously developed by Xi et al. [47]. Necessary modification to the front oral cavity was made to smoothly connect it to the MDI mouthpiece. The right upper panel in Figure 2a shows the mid-plane of the flow domain in the MDI and oral cavity, with unsymmetrical passages in the front and back of the MDI and a sudden expansion from the MDI mouthpiece to the oral cavity. To calculate regional drug deposition, the model was separated into mouth, pharynx, larynx, TB (tracheobronchial region), and five lung lobes: LU (left upper), LL (left lower), RU (right upper), RM (right middle), and RL (right lower), as shown in Figure 2b. The computational mesh was subsequently generated for the model geometry meshed for numerical analyses (Figure 2c).

**Figure 3:**
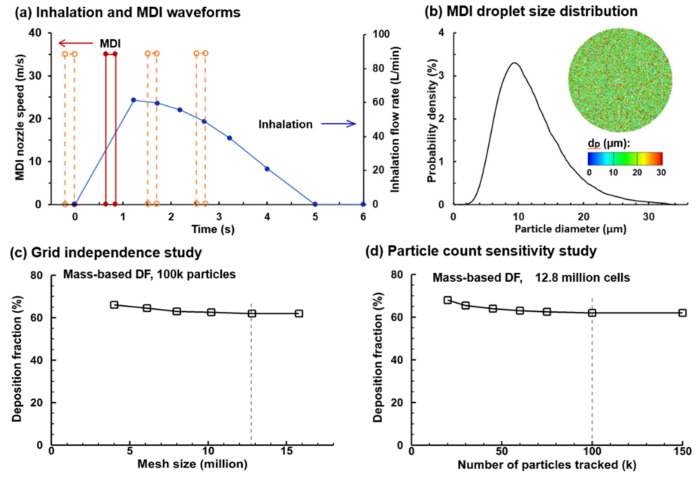
MDI delivery parameters and sensitivity studies: (**a**) deep slow inhalation and MDI administration waveforms; (**b**) MDI droplet size distribution, with a mean diameter of 11.0 µm and a geometric standard deviation of 1.57; (**c**) grid independence study with the mesh size ranging 4–16 million; and (**d**) droplet count sensitivity study with the number of sample droplet ranging 20–150 k. To study the effect of the MDI actuation timing on drug delivery, the MDI was applied at three more points within the inhalation cycle (i.e., 0.0, 1.5, and 2.5 s after inhalation onset) were considered in addition to the baseline case (i.e., 0.63 s after inhalation onset, red solid line).

In the control case, the MDI was actuated during 0.63–0.83 s after the onset of inhalation [39]. To study the effect of the MDI actuation timing (device–patient coordination) on the drug delivery, the MDI was applied at three more points within the inhalation cycle (i.e., 0.0, 1.5, and 2.5 s after the onset of inhalation; dotted brown lines, Figure 3a) in addition to the control case (i.e., 0.63 s after inhalation onset; red solid line, Figure 3a). The effects of actuation timing on drug dosimetry were assessed on both the regional and local basis.

#### 2.2.2. Measurement-Based Aerosol Size Distribution

An accurate MDI releasing model provides critical boundary conditions to ensure reliable CFD simulations of drug delivery. These include the size distribution of MDI droplets and their exiting speed at the MDI orifice. The aerosol droplets discharged from the orifice had a polydisperse size distribution followed the measurements by Liu et al., who quantified an albuterol inhaler by using a laser light scattering (LLS) system (Sympatec HELOS) [30]. The volume median diameter (Dv50) of the MDI cloud was 11.0 µm and the geometric standard deviation (GSD) was 1.57, with the lower bound being 0.5 µm and upper bound being 35.0 µm, as shown in Figure 3b.

#### 2.2.3. Mesh and Aerosol-Count Sensitivity Analysis

Sensitivity analyses were performed for both the computational mesh and seed droplet count to attain mesh-independent and statistically significant results (Figure 2c). Six mesh sizes were tested, ranging from 4.0 million to 16.0 million, and the mesh-independent deposition results were obtained at 12.6 million. Seven groups of seed droplets comprising 20, 30, 45, 60, 75, 100, and 150 k droplets, each with a distribution of Dv50 = 11.0 µm and GSD = 1.57 were tested, and a stable value was reached at 100 k and above (Figure 3d). As a result, a group of 100 k seed droplets was discharged from the orifice during MDI actuation.

#### 2.2.4. Determining the Droplet Discharging Velocity at the Orifice

**Figure 4:**
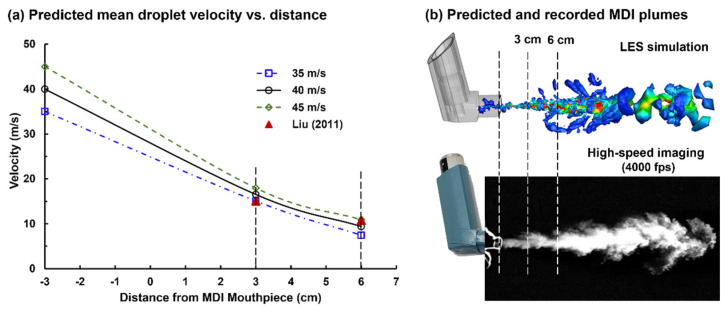
Estimating the droplet exiting velocity at the nozzle based on experimental measurements at downstream locations: (**a**) numerically predicted mean droplet velocities at 3 and 6 cm from the nozzle for three nozzle speeds (35, 40, and 45 m/s) in comparison to the phase Doppler anemometry (PDA) measurements [30]; (**b**) comparison between the numerically predicted and recorded MDI plumes.

Liu et al. measured the MDI spray velocities at 3 and 6 cm downstream of the mouthpiece, using phase Doppler anemometry (PDA) [30]. Simulations were conducted to reversely identify the initial velocity at the nozzle orifice, with which the numerically predicted velocities at 3 and 5 cm could match the measured values. Figure 4a shows the velocity decay of MDI droplets for three initial velocities (35, 40, and 45 m/s). It is observed that the LES-predicted velocity decay rate is slightly faster than the PDA measurements from 3 to 6 cm. An overall good agreement was achieved when the initial MDI droplet velocity was 40 m/s, with the predicted velocity marginally over- and underestimating the measurement at 3 and 6 cm, respectively. The LES predicted MDI nozzle flows also compared favorably with the MDI plume images recorded by using the Phantom VEO high-speed camera (Figure 4b). Thus, 40 m/s was adopted in the subsequent simulations for both the flow and droplet initial speeds at the nozzle of the CFC-propelled albuterol MDI.

### 2.3. Flow and Aerosol Dynamics of the Control Case (Triggering at 0.63 s)

#### 2.3.1. Aerosol Discharging from the Orifice: 0.5–3.0 ms after Actuation Onset

**Figure 5:**
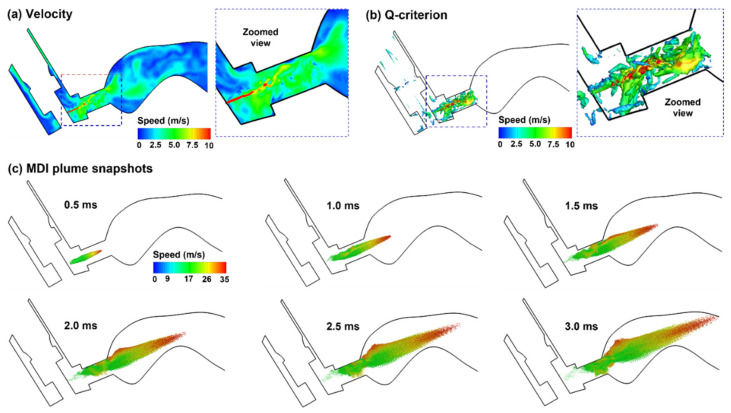
Airflow and droplet dynamics during MDI actuation (0.5–3.0 ms): (**a**) mid-plane velocity contour showing the jet flow from the nozzle and the heterogeneous flow distribution in the oral cavity, (**b**) jet-induced vortex structures in the mouthpiece of the MDI, and (**c**) snapshots of the MDI plume at varying instants (0.5–3.0 ms) after actuation. Flow and droplets were colored by their perspective velocities.

The flow and droplet dynamics immediately after the actuation (0.5–3.0 ms) are shown in Figure 5 for the control case (actuation at 0.63–0.8 s after inhalation onset). The jet flow from the orifice persisted for a short distance (3.0 mm) within the mouthpiece and then quickly merged with the inhalation flow. Due to the transient nature in both MDI discharge and inhalation maneuver (deep slow), quickly evolving flows with complex patterns are noted inside the MDI and oral cavity (Figure 5a). The jet flow from the orifice induced a trail of vortices within the mouthpiece, which quickly decayed in the oral cavity (Figure 5b). Figure 5c shows the snapshots of the MDI spray plume at different instants after actuation (i.e., 0.5–3.0 ms). Note the difference in the range of the color bars between the airflow (0–10 m/s) and aerosols (0–35 m/s), indicating that aerosol droplets maintain their momentum better than airflows. Droplets are observed to reach the back of the throat at 3.0 ms. Due to inertia, large droplets sustain their speeds longer and travel faster than small droplets, thus reaching the back throat earlier. This is consistent with the cold sensation in the oropharynx during MDI actuation [1,48]. During this short period (0–3 ms), the spray plume still looks focused, reflecting the dominant convective mechanism during MDI actuation.

#### 2.3.2. Aerosol Dispersion in the Upper Airway: 20–60 ms after Actuation Onset

**Figure 6:**
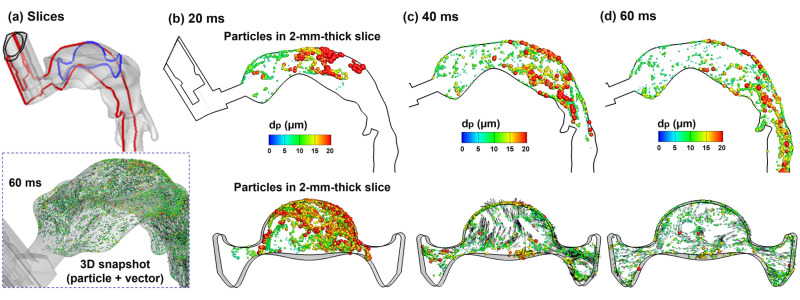
Droplet dynamics within the oral cavity 20–60 ms after MDI actuation within a 2 mm–thick slice: (**a**) two sampling locations, namely a sagittal slice (red, mid-plane) and an axial slice (blue); and snapshots of droplets in the sagittal and axial slices at three instants: (**b**) 20 ms, (**c**) 40 ms, and (**d**) 60 ms. Symbols of droplets were colored and scaled by their sizes.

Droplet transport in the upper airway is illustrated in Figure 6 in selected slices at three sequential times. Each slice has a uniform thickness of 1 mm, and only the droplets inside the slice at that instant are visualized; otherwise, droplets will fill the entire oral airway with no obvious pattern in a stationary image. More comprehensive visualization of droplet behaviors can be viewed in the Supplementary Materials Video S1 (upper airway) and Video S2 (mouth–lung), which shows the dynamic evolution of all droplets (aerosol-cloud) with a high time resolution. Very complex aerosol distributions are found both spatially and temporally (Figure 6b and Supplementary Materials Video S1). Due to the sudden expansion from the mouthpiece to the oral cavity, slow-moving and recirculating droplets are found in the circumferential region of the oral cavity. When holding the MDI mouthpiece, the oral cavity has a relatively large height between the tongue and mouth roof, and thus acts as a reservoir for MDI aerosols. Large high-speed droplets mix with small low-speed droplets, forming a highly dispersed aerosol cloud in the oral cavity, which will be carried into the lung by the inhalation flow (Supplementary Materials Video S2). Note that the droplet initial velocities are much higher than those of the background flow.

#### 2.3.3. Aerosol Dispensing into the Lung: 200 ms after Actuation Onset

**Figure 7:**
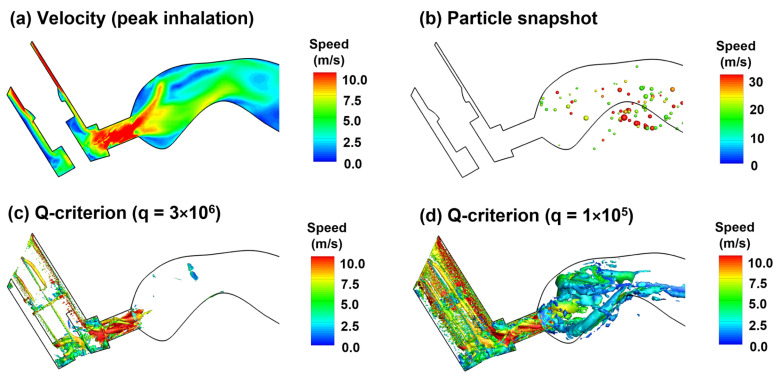
Airflow and droplet dynamics at peak inhalation (1.23 s, 61.2 L/min): (**a**) mid-plane 2D velocity contour, (**b**) snapshots of droplet positions, and instantaneous vortex structures defined by Q-criterion at two different levels (**c**,**d**).

With a deep slow inhalation waveform typical of MDI delivery, the peak flow occurs at around 0.6 s after MDI administration (or 1.23 after inhalation onset) at an approximate flow rate of 61 L/min. Considering the small area of the MDI mouthpiece (1.28 cm^2^), this flow rate gives rise to an average flow speed of 7.9 m/s (Figure 7a), which will quickly wash the aerosols suspending in the upper airway into the lung (Supplementary Video S2). Up to this point, most of MDI droplets have been deposited on the wall or conveyed to the lower airway, leaving only a small fraction of droplets that are trapped in recirculating flows. From Figure 7b, there are both small and large droplets lingering in the oral cavity, indicating the strong effect of local flow patterns (recirculation) on droplet behavior and fate in this region. The instantaneous vortex structures at the peak flow are displayed in Figure 7c,d, demonstrating strong vortices in the confined mouthpiece and weak vortices in the front half oral cavity, respectively.

#### 2.3.4. Aerosol Deposition

**Figure 8:**
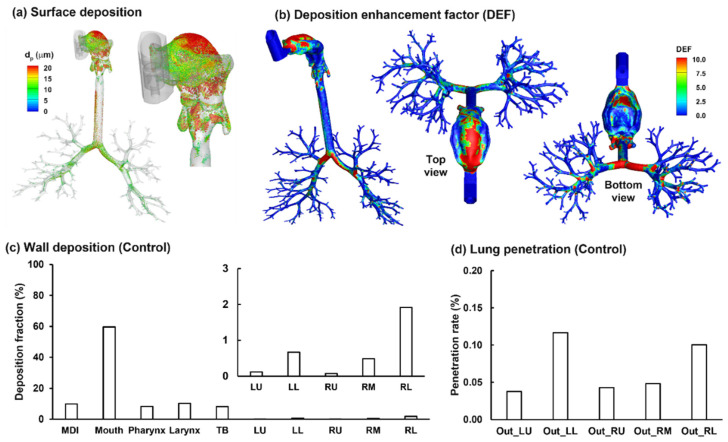
Characterization of the albuterol MDI delivery: (**a**) surface deposition with droplets scaled and colored by the droplet size, (**b**) dose distribution in terms of the deposition enhancement factor (DEF), (**c**) mass-based deposition fraction in different regions of the respiratory tract, and (**d**) drug fraction penetrating beyond the G9 bronchioles (i.e., the penetration rate).

The surface deposition of the polydisperse MDI droplets is displayed in Figure 8a. A salient feature is the size-dependent regional deposition. There is a significant deposition of large droplets (>17 µm, red color) in the back of the throat, due to the jet flow effect, as presented in Figure 5. By contrast, smaller droplets (<10 µm, green-blue color) are mostly found in the front half oral cavity and the lung. Most large droplets were deposited in the larynx and trachea, due to inertia impaction, particularly above the glottis aperture and in the main carina ridge.

Figure 8b shows the local deposition in terms of DEF, which is the ratio of the local deposition over average deposition [49]. Overall, the mouth is the major receiver of MDI-administered albuterol aerosols, highlighting the need to minimize the drug loss in this region. Deposition hot spots are noted on the roof of the oral cavity, the tip of the tongue, the main carina ridge, and, to a lesser intensity, the bifurcation ridges of subsequent branches (Figure 8b).

Regional deposition fractions (DFs) of albuterol drugs in different parts of the respiratory tract are shown in Figure 8c (left panel). It is noted that the albuterol droplets are polydisperse in size distribution, with a mean droplet diameter of 11 µm [30]; as a result, the DFs were calculated based on the deposited mass in contrast to the droplet count, as in most previous studies with monodisperse aerosols [50,51,52]. Again, the highest DF is found in the oral cavity (62.0%), followed by TB (11.9%), larynx (11.3%), and pharynx (9.5%). Including the MDI loss (1.3%), 96% of the albuterol is in the intrathoracic airways (mouth–TB), leaving only 4% for the thoracic regions. Considering this low availability, the DFs in the five lung lobes are presented at a scale of 0–3% (inset in Figure 8c). Much higher DFs were predicted in the lower lobes (i.e., LL and RL) than the upper lobes (LU and RU), presumably due to the droplet inertia and gravitational effects. The lung lobes in this study include branches from G2 to approximately G9 droplets exiting these bronchiolar outlets are considered as entering the deep lung (or pulmonary region). As shown in Figure 8d, even all at very low fractions, the lung penetration rates differ noticeably among the five lobes, with the lower lobes receiving much higher doses than those upper lobes (i.e., G10 and beyond).

### 2.4. Effect of Early and Delayed Actuation Times

#### 2.4.1. Airflow and Droplet Dynamics during 0.5–5 ms for 0 s Actuation

**Figure 9:**
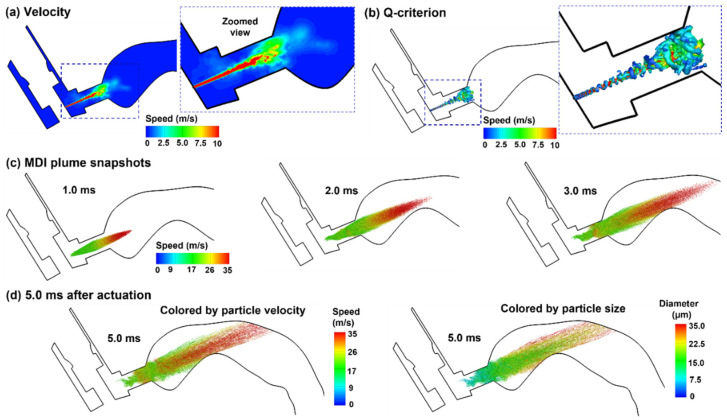
Airflow and droplet dynamics with MDI actuation before inhalation (t = 0.0): (**a**) mid-plane velocity contour showing the jet flow from the nozzle and the heterogeneous flow distribution in the oral cavity, (**b**) jet-induced vortex structures in the mouthpiece of the MDI, (**c**) snapshots of the MDI plume at varying instants (0.5–3.0 ms) after actuation with droplets colored by droplet velocity, and (**d**) comparison of the plume snapshots at 5.0 ms colored by droplet velocity and droplet size.

To investigate the effects of inhaler actuation timing on drug delivery efficiency, three more delivery scenarios (i.e., firing at 0.0, 1.5, and 2.5 s after the start of inhalation) were tested in addition to the control case (i.e., firing at 0.63 s). Appreciable differences in airflow and droplet dynamics were observed when actuating the inhaler at different points of the inhalation phase. For instance, when firing the inhaler into a quiescent oral cavity, the airflow and vortices were less turbulent than the control case (Figure 9a,b vs. Figure 5a,b). Without the transport of the secondary (inspiratory) flow, the droplets traveled a shorter distance for a given period and appeared less dispersed than the control case (Figure 9c vs. Figure 5c).

#### 2.4.2. Airflow and Droplet Dynamics during 20–400 ms for 0 s Actuation

**Figure 10:**
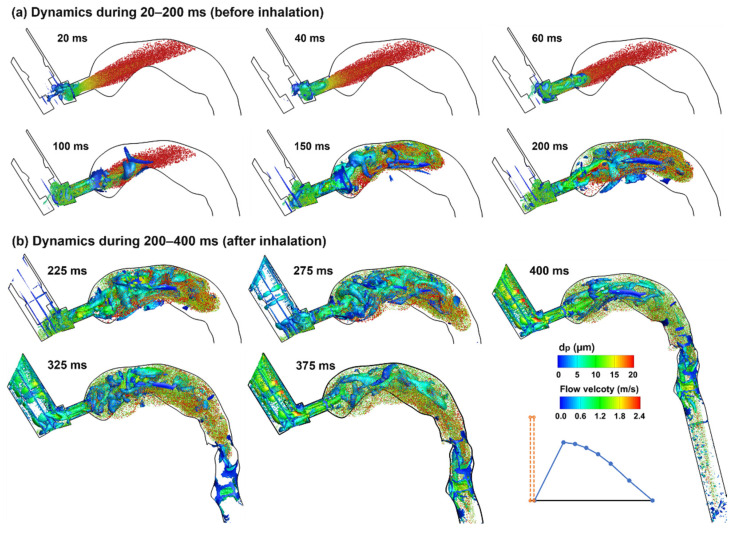
Droplet dynamics in the upper airway at varying instants (20–400 ms) when actuating the inhaler before inhalation: (**a**) dynamics during 20–200 ms, and (**b**) dynamics during 200–400 ms. Droplets were scaled and colored by droplet diameters. The Q-criterion iso-surfaces were colored by flow velocity.

Droplet dynamics in the second phase (20–200 ms) and third phase (200 ms and beyond) are shown in Figure 10a,b, respectively, for the case with MDI firing into quiescent airspace. Without an inhalation flow, the plume moved straight forward without apparent dispersion as opposed to the prompt dispersion in the control case. The large droplets (red color) quickly reduced their speeds, were floating between 20 and 100 ms, and began to settle down due to gravity. From 100 ms, the vortices caught up and the entrained small droplets (green color) mixed with large droplets in the back of the oral cavity. On the other hand, some droplets (small) re-entered the MDI mouthpiece (100–200 ms). From 200 ms, the inhalation started, and droplet dispersion became intensified. At the same time, droplets were transported by the inspiratory flow and advanced to the pharynx at 300 ms and the lower trachea at 400 ms.

#### 2.4.3. MDI Actuation Time Effects on Drug Dosimetry

**Figure 11:**
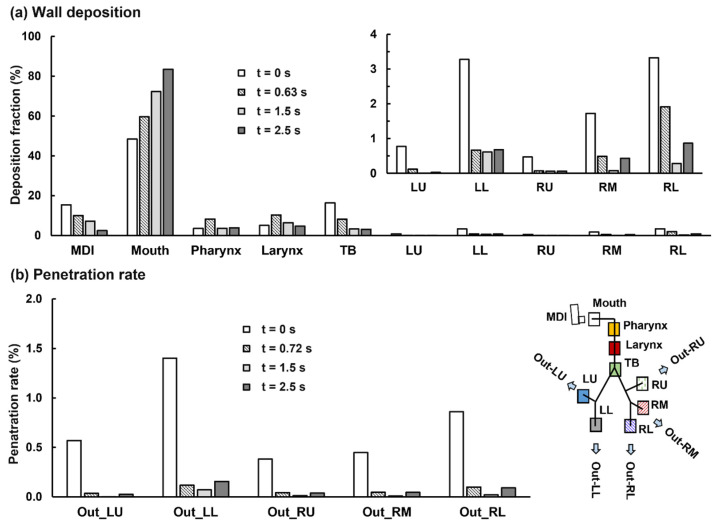
Comparison of the mass-based dosimetry between four delivery scenarios when the MDI was actuated at four different time-points of the inhalation waveform: (**a**) regional wall deposition and (**b**) lobar penetration rate.

Figure 11a compares the mass-based wall DFs between the four test cases (i.e., firing at 0, 0.63, 1.5, and 2.5 s). As expected, firing MDI at the late stages of the inhalation (i.e., t = 1.5 and 2.5 s) led to worse delivery efficiencies in most regions, including 12.5% and 23.8% more drug loss in the mouth and lower doses in the pharynx, larynx, and TB (Figure 9b).

To our surprise, firing MDI immediately before the inhalation gave rise to a lower DF in the mouth (i.e., 48.4% vs. 59.7% when firing at 0.63 s). Even though there is a higher loss in the MDI itself (15.4% when firing at 0.0 s vs. 10.0% at 0.63 s), this still leads to 5.9% more doses available for downstream airways. Relative to the control case, firing MDI at 0.0 s delivers persistently higher doses to the TB (control:16.4% vs. 8.2%) and all five lobes: LU (0.8% vs. 0.1%), LL (3.3% vs. 0.7%), RU (0.5% vs.0.1%), RM (1.7% vs. 0.5%), and RL (3.3% vs. 1.9%), as presented in Figure 11a. Considering that the frequent target of the albuterol MDI in asthmatic patients is the large airways (G0–G10), these improved doses predicted for 0 s MDI actuation have the potential to elicit better therapeutic outcomes, and thus definitely worth further investigations. The lobar penetration rates are also persistently higher when firing MDI at the beginning of the inhalation than the other three cases (Figure 11b). Even though the increase in the absolute penetration rate is not significant, due to the low drug availability to the deep lungs, the percentage increase is still very impressive. For instance, the penetration rate to the left lower deep lung (Out_LL, Figure 11b) is 1.4% vs. 0.1% in the control case, which only gives a 1.3% absolute increase, but a 1300% increase in percentage variation.

#### 2.4.4. MDI Actuation Time Effects on Deposition Distribution

**Figure 12:**
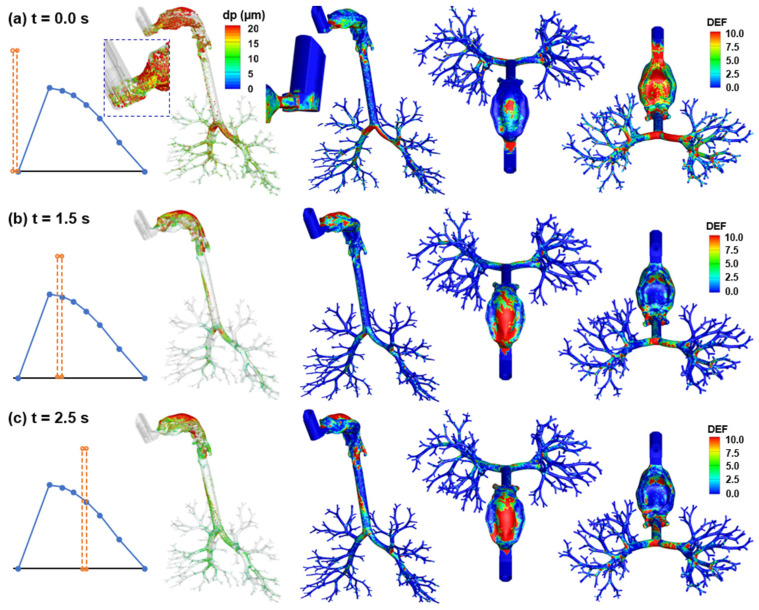
Comparison of surface deposition and DEF maps among three MDI actuation times: (**a**) firing at 0.0 s, (**b**) 1.5 s, and (**c**) 2.5 s.

Actuating the inhaler at different times led to very different deposition patterns. Larger droplets were deposited in the front mouth and MDI mouthpiece when firing at 0 s, while large droplets were mainly deposited in the back mouth when firing at 1.5 and 2.5 s (Figure 12). There is much higher deposition inside the MDI when firing at 0 s, while very few droplets were observed inside the MDI in the other two cases. The DEF distributions appear similar between t = 1.5 s and 2.5 s, likely owing to the similarities of airflows between these two cases (e.g., both passed the peak flow and were deaccelerating). Discrepancies between these two cases included elevated deposition in the lower trachea and the absence of large droplets (>15 µm) in the main carina ridge for 2.5 s actuation (Figure 12b vs. Figure 12c).

## 3. Discussion

In this study, MDI delivery of albuterol with CFC propellant was modeled in an integrated MDI–mouth–lung (G9) geometry with boundary conditions derived from experimental measurements. The effects of earlier and delayed MDI actuation relative to inhalation were evaluated on deposition in the device, oropharyngeal airway, and lung. High-speed imaging of the plume formation was recorded and compared favorably to complementary LES simulations.

### 3.1. Deposition Validation against Experiments

The predicted deposition fractions that use the LES–Lagrangian flow-droplet simulations provided a good approximation to comparable experimental tests of CFC MDI delivery. With prescribed press-and-breathe coordination, the drug loss in the device itself (i.e., 10%) reasonably matched the value measured in healthy volunteers (9.68 ± 1.90%) by De Backer et al. [53] and in a mouth–lung cast (6.4 ± 1.7%) by Cheng et al. [54]. The summative DF in the mouth, pharynx, and larynx herein (78.2% = 59.7% + 8.3% +10.2%) was close to the oropharyngeal–laryngeal DF (79.3 ± 1.9%) in Cheng et al. [54]. Similarly, the lung DF was 11.8% in this study vs. 13.5 ± 4.4% in Cheng et al. [54]. It is noted that this and Cheng’s studies utilized similar test conditions, with the same formulation (albuterol) and propellant (CFC) and a similar peak inhalation flow rate (~60 L/min). Moreover, the oral cavity in this study was originally modeled after that used in Cheng et al. [54]. The complexities of the lung models were also similar, with the computational lung model extending to G9, while the lung cast extended to G8 [54]. The close match between CFD and in vitro tests gave us confidence in subsequent evaluations of different delivery scenarios.

### 3.2. Three Phases of Aerosol Dynamics during MDI Delivery

A detailed examination of the spray droplet dynamics revealed that the press-and-breathe MDI delivery process can be divided into three distinct phases, regardless of the device actuation time. They are discharging (0–20 ms, from the orifice), dispersion (20–200 ms, in the oral cavity), and dispensing (>200 ms, to the lung). In the first phase, the jet effect from the nozzle orifice is dominant and the droplets have much higher speeds than the inspiratory airflows, leading to a spearhead-shaped aerosol cloud in the oral cavity. The cloud reduced its speed very quickly, and the cloud front (mainly composed of large droplets) reached the back of the throat at around 3.0 ms. During this phase, the droplet dynamics were almost independent of the inhalation flow. In the second phase, the droplet velocities became equivalent to the inspiratory flow, which was consistent with Oliveira et al., who observed a quick decrease in MDI droplet velocity from 40 m/s to the airflow velocity in a very short time (100 ms) [55]. The droplet behaviors in the upper airway were highly dependent on the local flows, where recirculation flows and vortices lead to intensified aerosol mixing and dispersion. As a result, we observed very different aerosol dynamics between the control and the t = 0 cases, with quick dispersion in the control case versus a much slower dispersion with device actuation at 0 s.

In the third phase, the floating, well-mixed aerosols in the oral cavity were entrained by the inhalation flow toward the lower airway. During this phase, droplet deposition mainly depended on the local velocity of the carrier flow (inertia) and the instantaneous aerosol concentration (availability). A higher loss in the throat was expected when a high concentration of aerosol passed the throat with a higher speed, and vice versa. Likewise, the lobar dose distribution depended on the instantaneous aerosol concentration, lobar ventilation, and droplet size; for small droplets, a slow lobar flow led to a relatively even distribution in lobar doses, while for large droplets, this led to unevenly higher deposition in the lower lobes. Large droplets would preferentially deposit in two lower lobes, due to gravity, while small droplets would deposit more uniformly among the five lobes when high-concentration droplets were inhaled slowly (i.e., with low inertia to closely follow the ventilatory flow).

### 3.3. Effect of Actuation–Inhalation Coordination on Deposition Distribution

Both early and delayed MDI actuation were considered in comparison to the control case (perfect coordination). Late device actuation relative to inhalation led to higher drug loss in the mouth and lower deposition in the pharynx, larynx, and TB (Figure 11a). Late actuation also led to overall lower lung doses in both the central (G2–G9) and peripheral (G10 and beyond) airways. However, this reduction was not significant compared to the variation in the upper airways. This was consistent with Pleasants et al., who observed certain, but not significant, (<25%) lung dose losses as long as the triggering was not later than 2.5 s.

As expected, triggering the device just before inhalation resulted in a higher loss in the device itself (15.4% vs. 10.0% in the control case, Figure 11a), mainly because of the counter flow from the mouth into the MDI mouthpiece. To our surprise, it also led to improved doses in nearly all airway regions. Compared to the control case, firing before inhalation decreased the drug loss in the mouth (from 59.6% in the control case to 48.4%), pharynx (from 8.3% to 3.5%), and larynx (from 10.3% to 5.0%), thus significantly increase the drug bioavailability to the lung, i.e., 15.9% more after deducting a 5.4% mouth loss). Furthermore, even though the absolute dose increases appeared to not be significant (i.e., 8.2% from 8.2 to 16.4% in TB), the percentage increase was high, i.e., 100% = (16.4–8.2%)/8.2%, as shown in Figure 11a. This percentage increase was more pronounced in the lower lobes than in the central and peripheral airways (Figure 11a,b). The underlying mechanisms that led to this improved delivery are deliberated below.

Even discharged at the same velocity from the orifice, the spray droplets were found to have a wide range of velocities shortly after their release (i.e., 10–35 m/s), with fast-moving droplets spearheading forward and impinging on the back wall of the throat, and slow-moving droplets lagging (Figure 9c). This velocity heterogeneity came from both the polydisperse size distribution and the intensified vortices that enhanced aerosol mixing. The inertia of a droplet is predominantly dependent on its diameter (with a power of 3) and velocity (with a power of 2); to evaluate their effects separately, the same aerosol clouds at 5.0 ms were plotted in two colors (Figure 9d), with one colored by the droplet velocity and the other by the droplet size. It is interesting to observe that the high-speed droplets are concentrated in the jet centerline, while the large droplets are more found in the plume periphery (Figure 9d). Small droplets can still avoid impacting on the back throat by closely following the curvature streamlines; by contrast, large droplets with high speeds are less likely to follow curvature streamlines. As a large droplet carries much larger mass than a small droplet (d^3^), any mechanism that can reduce the deposition of large droplets in the mouth has the potential to significantly improve the drug availability to the downstream airways. Triggering the device just before inhalation reduced the inertia enhancement from the accelerating inhalation flows. It also allowed more time for aerosol dispersion and delayed the aerosol transport to the lung, thus resulting in a lower oropharyngeal loss and higher lung doses. This was somewhat equivalent to adding a spacer, which could also decrease oropharyngeal loss and improve lung delivery, as demonstrated experimentally by Cheng et al. [54].

### 3.4. Limitations

There are several simplifications that were made in this study that might affect the result accuracy herein. These include a lung geometry extending to G9, rigid walls, isothermal condition, invariant droplet diameters (no evaporation), no electrostatic charge, and dilute concentration (no droplet collision/breakup/agglomeration). However, even with rapid advances in imaging technology, developing a lung network model with G9 bifurcations and beyond is still highly challenging. In particular, modeling of CT-based small airways and pulmonary alveoli can be more demanding, due to their inaccessibility, small size, complex structure, and dynamic nature [56]. During spray actuation, a large temperature gradient may exist in the near-nozzle region due to evaporation, which can change the droplet size and generate thermophoretic forces, thereby altering their deposition inside the device [57,58,59]. Considering the quick decay of droplet speeds during discharge and strong dispersion in the oral cavity, temperature gradient effects should only be important in the near nozzle region. In this study, the predicted MDI deposition in the control case (10%) agreed well with the in vitro measurement (6.4–9.7%) [53,54], indicating the nozzle orifice boundary condition that was reversely determined from complemental experiments did capture the dynamics of discharging droplets.

The lung geometry hereof was developed by using mathematical algorithms [60,61,62,63], but not from patient-specific CT scans that, once available, could be easily implemented in the current computational model. Electrostatic charges can occur during spray formation and have been demonstrated to affect droplet behaviors in both the near-wall region and core flows [64,65,66,67]. The respiratory tract expands noticeably during the slow deep inhalation in MDI delivery, which can also alter the flow and aerosol behaviors [68,69,70]. However, these factors can be secondary in comparison to those considered in this study, and neglecting these factors will not affect the numerical modeling results significantly. In this study, we did consider the major variables, such as a measurement-derived discharging velocity, an experimentally determined polydisperse size distribution, realistic actuation–inhalation waveforms, and flow-droplet heterogeneity in the central airway.

## 4. Materials and Methods

### 4.1. MDI–Mouth–Lung Model Development

The MDI model geometry was developed in SolidWorks based on an albuterol MDI. The oral cavity was based on an oral impression cast that was first reported by Cheng et al. in 1999 [71]. The pharyngolaryngeal region was reconstructed by segmenting CT scans of a 53-year-female, with detailed dimensions provided by Xi and Longest [72]. The lung network model was developed by using an in-house code *Lung4Cer* by Kitaoka et al. [60,61,62,63]. The lung geometry extended from the trachea to G9 (the ninth bifurcation generation) and consisted of 512 bronchiolar outlets. The trachea inclined forward by 15° and represented an upright body position.

### 4.2. High-Speed Imaging

The MDI actuation occurs within a very short duration. To understand the formation and transportation of the MDI spray plume, a high-speed camera (Phantom VEO, with the acquisition speed up to 11,000 fps) was used to record spatial and temporal evolutions of the aerosols exiting from the nozzle orifice at an acquisition rate of 4000 fps. A laser sheet (OXlaser, 488 nm, 100 mW) was utilized to increase the contrast between the aerosol cloud and the ambient air.

### 4.3. Numerical Methods

Isothermal and incompressible flows were assumed for the inhaled air. There were two airflows: the jet flow from the MDI nozzle orifice during device actuation and the patient’s inspiratory flow throughout the delivery process. The jet flow followed a step waveform that lasted 0.2 s and had the maximal velocity to be determined from experimental measurements (red line, Figure 2a). The inhaled airflow followed a waveform typically adopted for MDI applications (i.e., deep slow) and entered the airway through the space between the MDI holder and canister (blue line, Figure 2a). The inhalation lasted 5 s, with a peak flow rate of 61.2 L/min at 1.23 s after inhalation. One second of breath-holding was also simulated by following the inhalation maneuver to ensure all droplets either complete their deposition or exit the airway geometry.

To simulate drug delivery in the integrated MDI–mouth–lung model, a boundary condition at the MDI orifice is desired in terms of the flow and droplet discharge velocities. Liu et al. [30] measured the mean droplet velocities of an albuterol MDI inhaler, using a phase Doppler anemometry (PDA), and showed a 28.5% decrease in speed from 3 to 6 cm (i.e., from 15.1 to 10.8 m/s). To match the measured velocities, different initial droplet velocities were computed by using large-eddy simulations (LES) in order to reversely determine an appropriate nozzle boundary condition.

The LES–WALE approach was selected to solve the inhalation flows; this approach had been demonstrated to accurately capture the transitions between laminar and turbulent flow regimes [73]. A group of 100,000 droplets was discharged from the 0.5 mm–diameter nozzle orifice and their motions were tracked by using a discrete-phase Lagrangian method. Following the measurements by Liu et al. [30], the volume median diameter (Dv50) of the MDI cloud was 11.0 µm and the geometric standard deviation (GSD) was 1.57, with the lower bound being 0.5 µm and upper bound being 35.0 µm. A Matlab code was developed to generate the MDI-releasing droplets from the nozzle, which considered the polydisperse size distribution, variable exiting speeds, nozzle diameters, and plume angle. The airway walls were rigid and had a no-slip condition (i.e., u_wall_ = 0). Wall deposition was assumed whenever droplets touched the wall. A near-wall interpolation algorithm (NWI) was implemented, which had demonstrated improved predictions for both nano- and micro-meter aerosols [74].

Airflow fields and droplet motions were solved by using ANSYS Fluent 19.1 (Canonsburg, PA, USA). Mesh generation was performed by using ICEM CFD. Considering the multiscale feature of the model geometry (i.e., 0.5 mm diameter at the nozzle orifice, 40 mm width in the oral cavity, and 1.5 mm diameter at bronchiolar outlets), a multi-domain mesh was generated, with fine mesh downstream of the orifice and at the bronchiolar outlets, as well as coarse mesh in the upper airway (Figure 1). Prism elements were generated in the near-wall region to capture the boundary layer flows. An adequately fine mesh with body-fitted prism elements had been shown to play a crucial role in matching CFD predictions and comparable measurements for both nanoparticles [50,74] and micrometer particles [75].

With a 12.6-million mesh, 100 k seed droplets, a 0.0005 s time size, and a 6 s delivery time (5 s inhalation plus 1 s breath-holding), one test case took 280 h or so in an AMD Ryzen 9 3960x workstation with 3.79 GHz frequency and 256 G RAM. The drug dosimetry was characterized in three approaches: surface deposition, deposition fraction (DF), and deposition enhancement factors (DEF). Considering the polydisperse nature of MDI droplets, a mass-based DF in the region i of the respiratory tract was calculated as a post-processing procedure:

(1) $${\mathrm{DF}}_{i}=\frac{\sum_{j=1}^{N}\rho_{p}\left({\pi d}_{j}^{3}/6\right)}{\mathrm{Total}\;\mathrm{mass}\;\mathrm{of}\;\mathrm{seed}\;\mathrm{particles}}$$

Here j and N are the individual droplet index and the number of droplets deposited in the region i, ρ_p_ is the droplet density, and d_j_ is the droplet diameter. To quantify drug deposition distributions, the deposition enhancement factor (DEF) was computed as the ratio of the DF on a local area (i.e., a 0.5 mm–diameter circle) over the averaged DF [75]:

(2) $${\mathrm{DEF}}_{i}=\frac{{\mathrm{DF}}_{i}/A_{i}}{\mathrm{Total}\;\mathrm{mass}\;\mathrm{of}\;\mathrm{seed}\;\mathrm{particles}/\mathrm{total}\;\mathrm{area}\;\mathrm{of}\;\mathrm{the}\;\mathrm{airway}}$$

## 5. Conclusions

A computational model comparing an MDI, a mouth–throat, and a lung branching model to G9 was developed with experiment-based aerosol size distribution and discharging velocity. Airflow and droplet dynamics were examined in three different phases (i.e., discharging, dispersion, and dispensing) during the MDI delivery. The computational model was validated against in vitro measurements by Cheng et al. [54] under comparable test conditions. The validated model was further utilized to assess the impact of earlier and delayed device actuation on drug deposition distributions. Specific findings include the following:

1. The discharging velocity of the CFC-albuterol MDI aerosols was reversely determined to be 40 m/s to match the PDA measurements at 3 and 6 cm from the mouthpiece.
2. Highly trainset evolution of the MDI plume was recorded by using a high-speed camera at 4000 fps, which compared favorably with complementary LES simulations.
3. Good agreement was attained between CFD predictions and measured doses in the device, mouth–throat, and lung.
4. Delayed MDI triggering increased drug loss in the mouth and reduced doses in the lung.
5. MDI actuation just before the inhalation increased drug loss in the device, but led to improved dosimetry in the airway, including a reduced loss in the mouth–throat region and a higher delivery efficiency to the five lobes in both central and peripheral regions.

## Data Availability

The data presented in this study are available upon request from the corresponding author. The data are not publicly available due to privacy.

## Supplementary Materials

The following are available online at https://www.mdpi.com/article/10.3390/ph15010061/s1. Video S1: droplet dynamics in the upper airway. Video S2: droplet dynamics in the mouth–lung geometry.
